# Hypoxemic Respiratory Failure Due to Alveolar Pulmonary Sarcoidosis Following COVID-19 Infection

**DOI:** 10.7759/cureus.35790

**Published:** 2023-03-05

**Authors:** Hemanshi Y Mistry, Dominic Betancourt, Dan Schuller, Jonathan Lavezo

**Affiliations:** 1 Internal Medicine, The Hospitals of Providence - Transmountain/Texas Tech University Health Sciences Center El Paso, Paul L. Foster School of Medicine, El Paso, USA; 2 Internal Medicine/Pulmonary and Critical Care Medicine, The Hospitals of Providence - Transmountain/Texas Tech University Health Sciences Center El Paso, Paul L. Foster School of Medicine, El Paso, USA; 3 Pathology, The Hospitals of Providence - Transmountain/Texas Tech University Health Sciences Center El Paso, Paul L. Foster School of Medicine, El Paso, USA

**Keywords:** rapid progressive interstitial lung disease, case report, immune dysregulation, covid-19 infection, alveolar sarcoidosis

## Abstract

Pulmonary sarcoidosis is typically recognized as an interstitial lung disease with an infrequent occurrence of alveolar filling or acinar pattern. This rare form of alveolar sarcoidosis is known to have a rapid progression. Several case reports showed the development/worsening of sarcoidosis after COVID-19 infection. We present a case of a 60-year-old male with chronic hypoxic respiratory failure since having COVID-19 disease followed by gradual progression in symptoms, who had atypical sarcoid-like alveolar opacities on radiography, two prior negative bronchoscopies, transbronchial biopsy and BAL, and third bronchoscopic transbronchial biopsy suggestive of findings of poorly formed granulomas with high suspicion of alveolar sarcoidosis after ruling out other comparative possibilities, and later having a drastic improvement with sarcoidosis management. Our patient’s worsening symptoms after COVID-19 infection suggest impaired immunoregulation role of the infection in developing the disease process.

## Introduction

Sarcoidosis affects approximately 60 per 100,000 individuals in the US, with diffuse organ involvement [1]. The long-term complications (> four weeks) of COVID-19 infection are less well understood [2]. A few Chinese studies observed hospitalized COVID-19 patients for six months demonstrated ground glass opacities and consolidations as common residual CT findings, from which Han et al. found that 35% of patients had fibrotic and parenchymal changes like it is observed in sarcoidosis [3].

This article was previously presented as an oral presentation at the 2022 Annual Northwest ACP Clinical Vignette competition on May 6, 2022.

## Case presentation

A 60-year-old Caucasian male with a medical history of type 2 diabetes, hypertension, stage III anorectal carcinoma (s/p neoadjuvant chemoradiation therapy), and COVID-19 pneumonia followed by chronic hypoxemic respiratory failure (needing 2 L/min supplemental O_2_) nine months ago, presented to the emergency department with worsening dyspnea, non-productive cough, back pain, weight loss, lack of appetite and subjective cognitive decline over several months.

**Figure 1:**
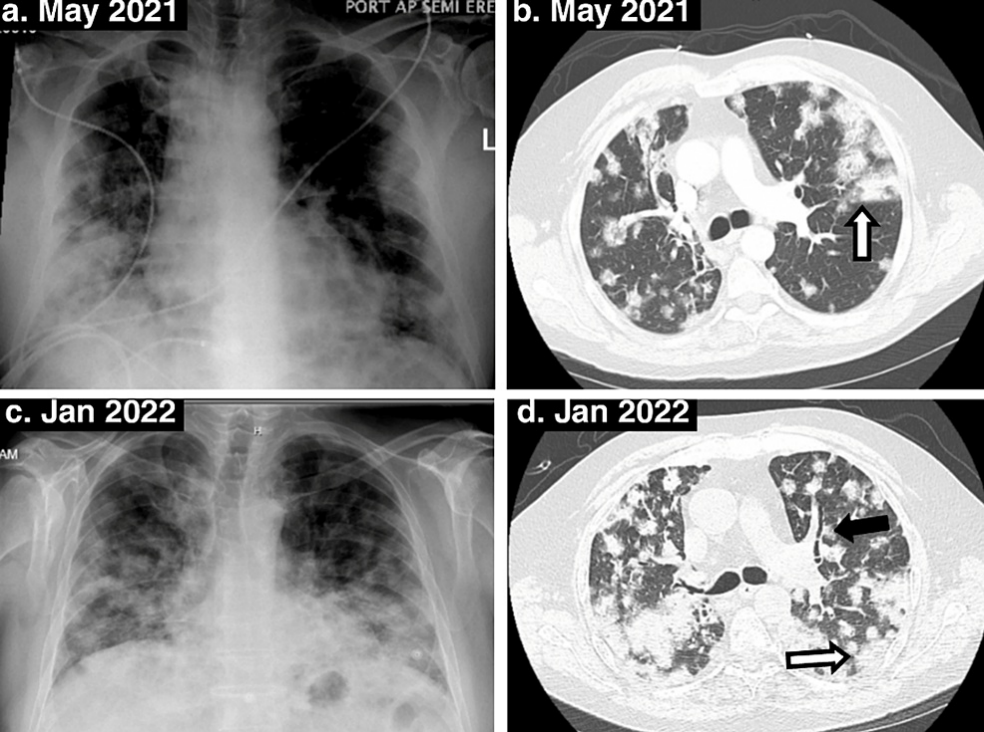
Initial and subsequent radiographic images demonstrating the progression of multiple, bilateral, irregular nodular densities with a predominantly peripheral distribution (white arrows) and air-bronchograms (black arrow), no pathologically enlarged hilar, mediastinal, or axillary lymphadenopathy.

Since COVID-19 pneumonia, he was noted to have persistent multi-lobar bilateral nodular opacities; on radiographs, we noted no hilar lymphadenopathy but parenchymal infiltrates and, thus, was categorized at Stage III of scadding radiographic stage (Figures 1a-1d); however, we could not localize imaging before that. For these findings, two outpatient bronchoscopies with transbronchial biopsies performed within nine months showed mixed acute and chronic interstitial inflammatory cells with negative BAL samples for AFB, bacterial, viral, and fungal cultures, negative cytology for malignancy and CD4:CD8 was not obtained for either of them and thus formally was never diagnosed with sarcoidosis. We do not have any prior vasculitis workup performed before this hospitalization.

On the current presentation, he had HR 109 bpm, BP 109/80 mmHg, respiratory rate 20/min, and saturating 83% on 3 L/min via nasal cannula. Diagnostic studies obtained on admission are displayed in Table 1. Pertinent elevated findings include creatinine, calcium, and Vitamin D 1-25OH. The patient was not on Vitamin D supplementation. Relevant decreased results include GFR, parathyroid hormone, and Vitamin D 25OH. SPEP, PTH-rap, and ACE levels were normal. Infectious screening for COVID-19 and Influenza both returned non-detectable. Chest x-ray (CXR) and high-resolution CT (HRCT) chest without contrast are explained in Figures 1a-1d. Further workup demonstrated normal TSH, AFP, CA 19-9, CEA, ANA, RA factor, anti-scleroderma antibodies, and c-ANCA and p-ANCA during the hospitalization in January 2022, which were negative; had negative skin Tuberculin test, and HIV screen. The patient's acute kidney injury later improved with fluid resuscitation.

**Table 1:** Laboratory value of current presentation compared with laboratory values on prior admission nine months ago N/A = Not Available eGFR = Estimated Glomerular Filtration Rate; TSH = Thyroid Stimulating Hormone PTH = Parathyroid Hormone; PTHrP = Parathyroid Hormone Related Protein ACE = Angiotensin Converting Enzyme ANA= Anti-Nuclear Antibodies c-ANCA= Antineutrophilic Cytoplasmic Autoantibodies, cytoplasmic p-ANCA= Perinuclear Anti-Neutrophilic Cytoplasmic Antibodies Anti SCL 70 antibodies= Anti Scleroderma 70 antibodies RA= Rheumatoid Arthritis factor *Random Urine Protein Electrophoresis (U PEP) Results: No monoclonal bands (M-spikes) seen in the gamma region. **Serum Protein Electrophoresis (S PEP) Results SPEP: No monoclonal bands (M-spikes) seen in the gamma region. COVID-19 = Coronavirus SARS CoV-2; NAA = Nucleic Acid Amplification; PCR = Polymerase Chain Reaction AFP = Alpha-Fetoprotein; CA 19-9 = Cancer Antigen 19-9; CEA = Carcinoembryonic Antigen

Initial Admission Diagnostic Testing			
Test Name	Current admission Value (01/2022)	Comparative value (05/2021)	Reference Range
Chemistry Panel			
Calcium (mg/dL)	13.7 (↑)	9.7	8.5-10.5
Creatinine (mg/dL)	1.9 (↑)	0.9	0.3 – 1.3
eGFR (mL/min/1.73m^2^)	36 (↓)	24	≥60
TSH (µIU/mL)	0.490	0.420	0.4 – 5.0
PTH (pg/mL)	6.4 (↓)	N/A	15.0 – 65.0
PTHrP (pmol/L)	< 2.0	N/A	< 2.0
Vitamin D 25 OH (ng/mL)	28.6 (↓)	N/A	30.0 – 99.0
Vitamin D 1-25 OH (pg/mL)	176.0 (↑)	N/A	19.9 – 79.3
ACE (units/L)	32	N/A	14.0 – 82
ANA Screen	Negative	Negative	Negative
c-ANCA (AU/mL)	<19	N/A	=<19
p-ANCA (units/mL)	0.01	N/A	0.00-0.22
Anti-Scl 70 antibodies (AU/mL)	<29	N/A	=<29
RA factor (IU/mL)	<15	N/A	<15
Serum and Urine Electrophoresis			
S PEP	See Legend*	N/A	
U PEP	See Legend**	N/A	
Infectious Etiology Screening			
COVID-19 NAA	Negative	Positive	Negative
COVID-19 PCR	Not Detected	Detected	Not Detected
Influenza A PCR	Not Detected	Not Detected	Not Detected
Influenza B PCR	Not Detected	Not Detected	Not Detected
HIV1 p24 Antigen Direct	Non-Reactive	N/A	Non-Reactive
HIV 1/2 Antibody Direct	Non-Reactive	N/A	Non-Reactive
Skin Tuberculin Test	Negative	N/A	Negative
Malignancy Screening			
AFP (ng/mL)	2.0	N/A	≤ 9.0
CA 19-9 (units/mL)	13.6	N/A	0.0 – 35.0
CEA (ng/mL)	2.6	N/A	≤ 3.0

**Figure 2:**
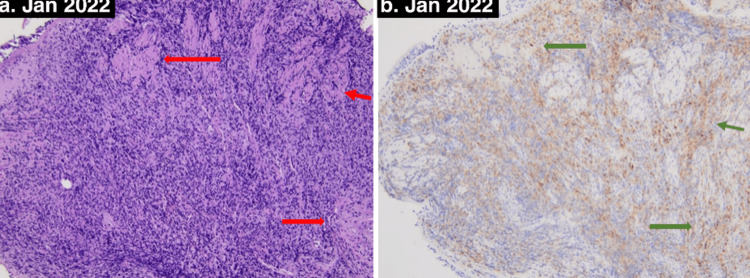
Diagnostic final transbronchial biopsy pathology results: (a) H&E stain demonstrated bronchial submucosa with extensive areas of chronic inflammation and focal aggregates of epithelioid histiocytes compatible with poorly formed non-caseating granulomas (red arrow). (b) Positive CD68 immunohistochemical stain confirming histocytes in areas of poorly formed non-caseating granulomas (green arrow).

**Figure 3:**
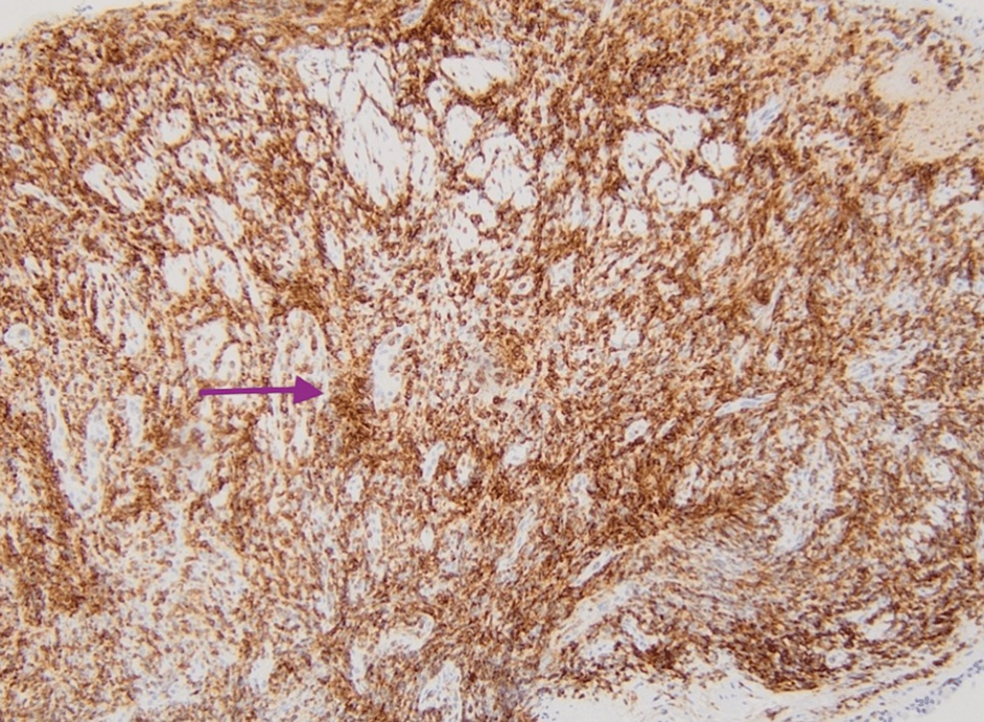
CD3 shows dense t-cell lymphocytic infiltrate (dark brown stains).

**Figure 4:**
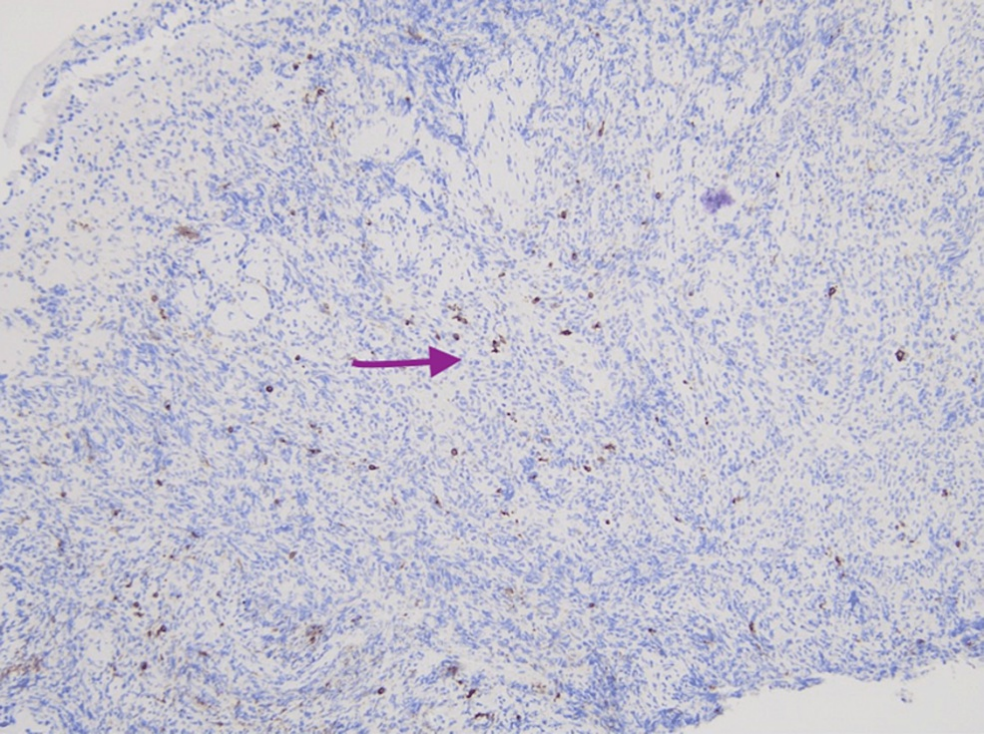
CD20 highlighted scattered B-cells (small dark brown dots).

A repeat CXR on hospital day 3 revealed interval worsening in the bilateral pulmonary opacities. A bronchoscopy was performed with bronchial washings, multiple fluoroscopically guided transbronchial brushings, and bronchoalveolar lavage (BAL) with no pathogens seen on stains and negative AFB, fungal and viral cultures and negative for malignancy. The pathology demonstrated extensive areas of chronic inflammation with epithelioid histiocytes, reactive pneumocytes, chronic interstitial inflammation, poorly formed non-caseating granulomas, no fibrosis, and negative cytology for malignancy. CD68 highlights admixed histiocytes (Figures 2a, 2b). CD3 highlights numerous T-cells in the submucosal lymphoid aggregate (Figure 3), and CD20 highlights occasional positive B-cells (Figure 4).

This rapid clinical presentation, hypercalcemia, radiographic images, poorly formed granulomata, and positive CD68 histiocytes by immunostaining were suspected of having a diagnosis of alveolar sarcoidosis. He was started on methylprednisolone 60mg IV q6hr, which led to a significant improvement in clinical status within three days, further reinforced our diagnosis, and was discharged home on hospital day fifteen with a continuous treatment regimen of prednisone 60 mg oral daily with a follow-up in the outpatient setting.

**Figure 5:**
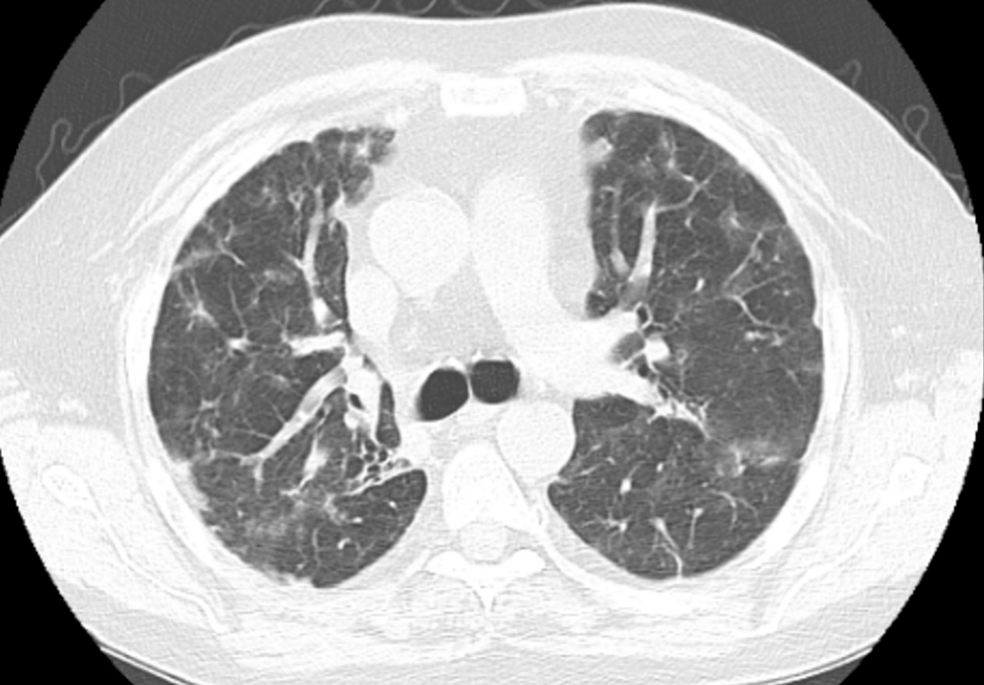
Follow-up CT chest with contrast two months after initiation of steroid therapy demonstrated marked improvement of the previously noted confluent and nodular opacities.

A 2-month post-hospitalization follow-up HRCT on prednisone (Fig 5) showed marked improvement in nodular opacifications, with significant clinical improvement. He did report steroid-induced side effects, including iatrogenic Cushing syndrome, oral thrush, weight gain, worsening hypertension, hyperglycemia, and ankle edema. He was started on methotrexate 7.5mg 1x/week as a steroid-sparing agent and a gradual tapering of prednisone with continued improvement. PET/CT was not obtained since starting methotrexate.

## Discussion

Sarcoidosis is a multisystem disorder that can affect practically any body organ, of which the lungs and intrathoracic lymph nodes are the most common (over 90% of patients). The annual incidence of sarcoidosis varies across ethnic groups, with the highest incidence observed among African Americans (17-35 per 100,000) and a slight female predominance which is entirely the opposite compared to our patient, in addition to his other atypical imaging and histopathologic features [4]. Alveolar sarcoidosis is a rare and rapid variant of sarcoidosis occurring in 1%-4% of patients. Typically, it has bilateral-multifocal, mass-like confluent and nodular opacities of >30 mm size, irregular border, air bronchogram, progressive architectural destruction, and fibrosis [5]. Bronchoscopy with transbronchial biopsies has a high diagnostic yield (>80%) for sarcoidosis; our case might have had poor parenchymal sampling in the prior two studies. Thus, it is difficult to say whether our patient already had sarcoidosis before having COVID-19 infection.

The probable diagnosis of alveolar sarcoidosis in our case was suggested by severe hypoxemia, rapid progression of multiple ill-defined pulmonary nodules, histopathology demonstrating chronic inflammation with poorly formed granulomata with negative vasculitis workup, and no risk factors of hypersensitivity pneumonitis, and the laboratory results significant for hypercalcemia, elevated 1, 25 OH vitamin D, along with his clinical and radiographic response to steroid treatment.

The pathophysiology of sarcoidosis is still a very challenging and dynamic topic, possibly related to environmental exposures, occupational insults to the pulmonary microenvironment, epigenetics, and familial clustering. A case report of a sarcoid patient with COVID-19 revealed increased CCR6+ Th17 in CD4 T cells. Considering the changes in the T-cell populations and levels of TH17-related cytokines, interleukin (IL) - 17, and interleukin-22, groups have postulated that sarcoid is a fundamentally immune-mediated response to an antigen that may be environmental or infectious [6,7]. We speculate that ours might be an example of SARS-CoV-2 infection that may predispose patients to rapid progression of pulmonary sarcoidosis via shared mechanisms of immune dysregulation; however, this requires further studies to connect them.

Systemic therapy should be reserved only for patients with progressive, symptomatic disease, persistent pulmonary infiltration, and progressive lung function decline [1]. First-line treatment for pulmonary sarcoidosis includes systemic glucocorticoids, specifically prednisone 20 to 40 mg/d for one to three months before gradual taper if radiographic, clinical, and PFT results are stable or improved. However, in refractory or recurrent cases, glucocorticoid-sparing agents (Methotrexate 10 to 25 mg weekly, oral or intramuscular) and biological agents (Azathioprine and Hydroxychloroquine) may be considered [8]. Routine screening for annual calcium, creatinine, and alkaline phosphatase is recommended after negative baseline screening to look for extrapulmonary involvement [9].

## Conclusions

Alveolar sarcoidosis is a less-studied type of pulmonary sarcoidosis. Its rapid progression and typical radiographic findings significantly distinguish it from the interstitial type of sarcoidosis. The pathophysiology of sarcoidosis is still a very challenging topic with many possible etiologies. This case highlights a novel role of inflammatory markers and T-cell-mediated immune dysregulation in COVID-19 infection and sarcoidosis, further strengthening their connection. Additional studies are needed to delineate the role of COVID-19 disease in either development or worsening of pulmonary sarcoidosis. Alveolar sarcoidosis should be considered in patients with non-improving post-COVID-19 syndrome symptoms, especially when findings suggest interstitial lung disease.
